# COVID-19 positive cases among asymptomatic individuals during the second wave in Ndola, Zambia

**DOI:** 10.4102/ajlm.v12i1.2119

**Published:** 2023-05-31

**Authors:** Jonathan Gwasupika, Victor Daka, Justin Chileshe, Moses Mukosha, Steward Mudenda, Bright Mukanga, Ruth L. Mfune, Gershom Chongwe

**Affiliations:** 1 Department of Clinical Sciences, Tropical Diseases Research Centre, Ndola, Zambia; 2 Department of Public Health, School of Medicine, Copperbelt University, Ndola, Zambia; 3 Department of Biomedical Sciences, Tropical Diseases Research Centre, Ndola, Zambia; 4 Department of Pharmacy, School of Health Sciences, University of Zambia, Lusaka, Zambia; 5 Tropical Diseases Research Centre, Ndola, Zambia

**Keywords:** asymptomatic individuals, COVID-19 disease, positivity rate, SARS-CoV-2, Zambia

## Abstract

**Background:**

Coronavirus disease 2019 (COVID-19) is a worldwide public health concern for healthcare workers. About 80% of cases appear to be asymptomatic, and about 3% may experience hospitalisation and later die. Less than 20% of studies have looked at the positivity rate of asymptomatic individuals.

**Objective:**

This study investigated the COVID-19 positivity rates among asymptomatic individuals during the second COVID-19 wave at one of Zambia’s largest testing centre.

**Methods:**

This was a retrospective cross-sectional study conducted on routine surveillance and laboratory data at the Tropical Diseases Research Centre COVID-19 laboratory in Ndola, Zambia, from 01 December 2020 to 31 March 2021. The study population was made up of persons that had tested for severe acute respiratory syndrome coronavirus 2 (SARS-CoV-2) infection as a requirement for travel. Microsoft Excel was used to come up with an epidemiological curve of daily COVID-19 positive cases; proportions for gender were described using frequencies and percentages.

**Results:**

A total of 11 144 asymptomatic individuals tested for SARS-CoV-2 were sampled for the study and 1781 (16.0%) returned positive results. The median age among those tested was 36 years (interquartile range: 29–46). Testing for COVID-19 peaked in the month of January 2021 (37.4%) and declined in March 2021 (21.0%). The epidemiological curve showed a combination of continuous and propagated point-source transmission.

**Conclusion:**

The positivity rate of 16.0% among asymptomatic individuals was high and could imply continued community transmission, especially during January 2021 and February 2021. We recommend heightened testing for SARS-CoV-2 among asymptomatic individuals.

**What this study adds:**

This study adds critical knowledge to the transmission of COVID-19 among asymptomatic travellers who are usually a key population in driving community infection. This knowledge is critical in instituting evidence-based interventions in the screening and management of travellers, and its control.

## Introduction

Coronavirus disease 2019 (COVID-19), caused by the severe acute respiratory syndrome coronavirus 2 (SARS-CoV-2), is a worldwide public health concern for healthcare workers, including physicians, public health specialists and researchers.^1^ The World Health Organization declared the outbreak of COVID-19, which was first reported in Wuhan, China, as a Public Health Emergency of International Concern on 30 January 2020,^2^ as it was posing a high risk to countries with vulnerable health systems.^3^ Almost all 55 countries in Africa have been affected by the coronavirus pandemic, with sub-Saharan Africa being the most affected.^4^ Zambia recorded its first case of COVID-19 on 18 March 2020^5^ and has since recorded a total of 333 555 COVID-19 cases and 4017 deaths as of 04 October 2022.^6^

Severe acute respiratory syndrome coronavirus causes a number of human respiratory disease conditions, ranging from mild cold to severe respiratory distress syndrome, and it largely spreads between persons by respiratory droplets and contact routes.^7^ On the other hand, SARS-CoV-2 has been seen to spread faster than SARS-CoV, which was first reported in 2003 and caused previous outbreaks. Accumulating evidence showed that SARS-CoV-2, unlike SARS-CoV, is transmitted by persons without symptoms.^8^ About 80% of cases appear to be asymptomatic,^4,9,10^ and about 3.3% may experience hospitalisation and later die. Based on global biological, epidemiological and modelling evidence, asymptomatic COVID-19 may play a substantial role in the pandemic trajectory.^11^

Despite public health preventive measures such as hand hygiene, social distancing, quarantine and travel restrictions which were instituted, transmission of COVID-19 seemed to be ongoing.^12^ Vaccination against COVID-19 has been shown to be effective against contracting severe forms of the disease.^13^ However, vaccination does not protect an individual from transmitting or becoming infected with SARS-CoV-2.^14^

Zambia has experienced four waves of COVID-19.^1^ During the first wave to about the fourth wave of COVID-19, it was a requirement to have a negative polymerase chain reaction COVID-19 certificate by everyone intending to travel.^15,16^ This study aimed to assess the positivity rate among asymptomatic travellers during the second wave of the COVID-19 pandemic, and its determinants.

## Methods

### Ethical considerations

Ethical approval to carry out the study was obtained from the TDRC Research Ethics Committee (IRB registration number: 00002911). Permission to carry out the study and access to patient information was obtained from the Tropical Diseases Research Centre management. Informed consent was not obtained from any individual as there was no active participation in the study. Confidentiality of patient information was adhered to and data were de-identified prior to analysis.

### Study design and site

This was a retrospective cross-sectional study conducted on surveillance and laboratory data collected at the Tropical Diseases Research Centre (TDRC) COVID-19 laboratory in Ndola, Zambia, from 01 December 2020 to 31 March 2021. The TDRC is a national health research institution specialising in both infectious and non-infectious diseases. The TDRC COVID-19 laboratory is accredited for certification of travellers by the African Society for Laboratory Medicine and conducts approximately 400 COVID-19 tests per day.

### Study population and eligibility criteria

The study population was made up of persons who were tested for SARS-CoV-2 infection during the second wave of COVID-19. Complete enumeration of the data set comprising individuals tested for COVID-19 was obtained for analysis. Eligibility for testing was based on getting tested for COVID-19 as a mandatory requirement for international travel, regardless of age. Tests of individuals that were collected from outside the TDRC and those that were done outside the stipulated period of the second wave were not included in the analysis. Additionally, tests that were done after vaccination had begun were excluded.

### Data collection

Data were collected from an already-prepared Microsoft Excel spreadsheet (Microsoft Corporation, Redmond, Washington, United States) and a case investigation form comprising the following information: date the test was done, age, gender, and results. An extraction data tool was used for data collection. Variables with no clear labels and missing data were removed from the data set.

### Data analysis

Microsoft Excel was used to come up with an epidemiological curve of daily COVID-19-positive cases, whereas proportions for gender were described using frequencies and percentages. Age was described as a continuous variable and the mean, median, mode and range were used. To test for differences on the COVID-19 test result, the chi-square test was used once assumptions were met to analyse binary variables; otherwise Fisher’s exact test was used. For continuous variables; the Mann-Whitney ranksum test was used for skewed data. After stratifying COVID-19 positivity by months, the one-way analysis of variance test was used to analyse for differences in age among groups. To predict factors associated with a positive test for COVID-19, logistic regression methods were used. STATA^®^ software, version 14 SE (STATA Corp., College Station, Texas, United States) was used for analysis. A *p*-value less than 0.05 was considered statistically significant at a confidence interval of 95%.

## Results

A total of 11 144 asymptomatic travellers tested for COVID-19 were sampled for the study and 1781 tested positive, resulting in a positivity rate of 16.0% (Table 1). The study participants had a median age of 36 years (interquartile range: 29 to 36 years). The test for COVID-19 was noted to be done mostly by travellers in the age group 19 to 50 years. The youngest participant was 1 year old while the oldest was 92 years old. A majority of those tested were male travellers (73.2%; 8152/11 144); female travellers accounted for the remaining 26.8% (2992/11 144). The highest number of tests were completed in January 2021 (4170/11 144).

**TABLE 1:** Basic characteristics of participants, Ndola, Zambia, December 2020 – March 2021.

Variable	Category	Frequency (*n*)	Percentage (%)	Median	IQR
**Age (years)**		-	-	36	29–46
	≤ 18	632	5.7	-	-
	19–50	8744	78.4	-	-
	≥ 50	1768	15.9	-	-
**Gender**					
	Male	8152	73.2	-	-
	Female	2992	26.8	-	-
**Residence**					
	Ndola	8780	78.8	-	-
	Outside Ndola	2364	21.2	-	-
**Swab collection site**					
	Nasopharyngeal	11 107	99.6	-	-
	Oropharyngeal	37	0.4	-	-
**Month**					
	December 2020	1557	14.0	-	-
	January 2021	4170	37.4	-	-
	February 2021	3079	27.6	-	-
	March 2021	2338	21.0	-	-
**COVID-19 result**					
	Positive	1781	16.0	-	-
	Negative	9363	84.0	-	-

COVID-19, coronavirus disease 2019; IQR, interquartile range.

The proportion of female travellers that tested positive for COVID-19 (18.4%) was greater than the proportion of male travellers (15.1%) with a *p-*value of 0.027 (Table 2). Among individuals who tested for COVID-19 prior to travelling, about 7303 (83.2%) tested negative and were Ndola residents, whereas among individuals that tested positive, 304 (12.9%) were not Ndola residents. There were no positive results among individuals whose samples were collected orally. Of the monthly tests done, 30/1557 (1.9%) were positive in December 2020, 1060/4170 (25.4%) were positive in January 2021, 532/3079 (17.3%) positive in February 2021 and 159/2338 (6.8%) positive in March 2021.

**TABLE 2:** COVID-19 positivity rate and socio-demographics of participants in Ndola, Zambia, December 2020 – March 2021.

Variable	COVID-19 results				*p*-value
	Negative		Positive		
	*n*	%	*n*	%
**Age**	-	-	-	-	0.027*
≤ 18 years	551	87.2	81	12.8	-
19 to 50 years	7309	83.6	1435	16.4	-
≥ 50 years	1503	85.0	265	15.0	-
**Gender**	-	-	-	-	< 0.001*
Male	6913	84.9	1231	15.1	-
Female	2442	81.6	549	18.4	-
**Residence**	-	-	-	-	< 0.001*
Ndola	7303	83.2	1477	16.8	-
Outside Ndola	2060	87.1	304	12.9	-
**Sample collection site**	-	-	-	-	0.004**
Nasopharyngeal swab	9327	84.0	1781	16.0	-
Oral swab	36	100.0	0	0.0	-
**Month**	-	-	-	-	< 0.001*
December 2020	1527	98.1	30	1.9	-
January 2021	3110	74.6	1060	25.4	-
February 2021	2547	82.7	532	17.3	-
March 2021	2179	93.2	159	6.8	-

COVID-19, coronavirus disease 2019.

* , Pearson’s chi-square;

** , Fisher’s exact test.

When stratified by month of visit to the testing centre, there were more Ndola residents seeking testing services at the TDRC laboratory in January 2021 (*n* = 948) than any other month included in the study (Table 3). An equal peak number of non-Ndola residents (*n* = 111) was seen in January 2021 and February 2021. The lowest number of travellers was seen in December 2020.

**TABLE 3:** COVID-19 positivity rate stratified by months in Ndola, Zambia December 2020 – March 2021.

Variable	Period								*p*
	December 2020		January 2021		February 2021		March 2021		
	*n*	%	*n*	%	*n*	%	*n*	%
**Residence**	-		-		-		-		< 0.001*
Ndola	28	1.9	948	64.2	420	28.4	81	5.4	-
Outside Ndola	33	1.9	111	36.5	111	36.5	77	25.3	-
**Gender**	-		-		-		-		0.027*
Male	20	1.6	731	59.4	354	28.8	126	10.2	-
Female	13	2.4	327	59.6	177	32.2	32	5.8	-

COVID-19, coronavirus disease 2019.

* , Pearsons chi-square.

**FIGURE 1:**
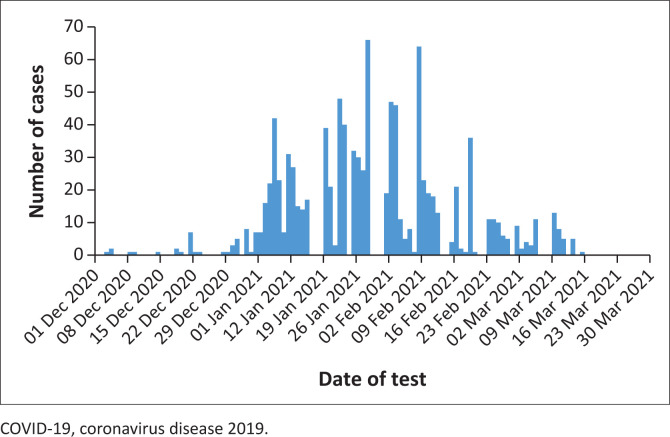
Daily COVID-19-positive cases in asymptomatic travellers in Ndola, Zambia, December 2020 – March 2021.

Coronavirus disease 2019 cases began to rise on 05 January 2021 and reached a peak on 26 January 2021 (Figure 1). The cases remained high until 23 February 2021, when there was a reduction of 150 in the number of positive cases reported.

Female travellers had a 16.0% (adjusted odds ratio: 1.16; 95% confidence interval: 1.03 – 1.30; *p* = 0.012) increased chance of testing positive for SARS-CoV-2 compared to male travellers, after adjusting for age, residence, and month in which the test was done (Table 4).

**TABLE 4:** Predictors of positive COVID-19 during the second wave in Ndola, Zambia, December 2020 – March 2021.

Variable	COR	*p*	95% CI	AOR	*p*	95% CI
**Age (years)**					
≤ 18	1.00	-	-	-	-	-
19 to 50	1.34	0.018	1.05–1.70	1.45	< 0.001	1.14–1.85
≥ 50	1.20	0.183	0.92–1.57	1.30	0.055	0.99–1.70
**Gender**					
Male	1.00	-	-	-	-	-
Female	1.26	< 0.001	1.13–1.41	1.16	0.012	1.03–1.30
**Residence**					
Ndola	1.00	-	-	-	-	-
Non-Ndola	0.73	< 0.001	0.63–0.83	1.08	0.284	0.93–1.26
**Month**					
December 2020	1.00	-	-	1.00	-	-
January 2021	18.4	< 0.001	12.6–26.9	18.31	< 0.001	12.5–26.8
February 2021	11.2	< 0.001	7.67–16.6	11.18	< 0.001	7.60–16.4
March 2021	3.93	< 0.001	2.61–5.90	3.81	< 0.001	2.51–5.78

Note: The factors that were adjusted for included; gender, age, month of doing a COVID test and residence.

95% CI, 95% confidence interval; AOR, adjusted odds ratio; COR, crude odds ratio; COVID-19, coronavirus disease 2019.

## Discussion

This study found a positivity rate of 16.0% (1781/11 144), with 69.1% (1231/1781) of male travellers being affected. The months of January 2021 and February 2021 recorded the highest rate of positivity. The epidemiological curve showed that the second wave of COVID-19 lasted from December 2020 to the end of March 2021. Further, the chances of testing positive for SARS-CoV-2 if an individual was female increased by about 16% (95% confidence interval: 1.03–1.30) compared to being male, after controlling for other variables.

The positivity rate found in this study was similar to the positivity rate at the national level in Zambia during the same period.^6^ Despite the similarity, the national level positivity rate comprised both symptomatic and asymptomatic cases. The positivity rate found could have been higher if control measures of isolation and quarantine of cases and testing of people before travel were not put in place and followed.^11^ On the other hand, the positivity rate in this study was higher than the national rate of 10.6% during the first wave.^15^ This could have been due to differences in the attack rate and rate of transmission of the strain of coronavirus.^16^ Conversely, a study in Nigeria reported a higher positivity rate of 20.8% in the second wave which lasted from 25 October 2020 to 03 April 2021, with asymptomatic cases being the majority.^17^ A study done by Avadhanula et al. showed a positivity rate of 11.4% among asymptomatic patients during the second wave between 18 March 2020 and 15 August 2020 in Houston, Texas, United States, which was much lower than the rate found in this study.^18^ This could have been due to differences in region, rate of transmission, adherence to recommended guidelines and utilisation of COVID-19 vaccine as it was introduced in some countries earlier than others.^17^ Our study and a study by Ghosh, Sarkar and Chouhan, done in India between March 2021 and May 2021, thus confirmed the presence of COVID-19 among asymptomatic individuals.^19^

Our study found that a rise in COVID-19 cases during the second wave of the pandemic was observed from December 2020 and ended in March 2021. The epidemiological curve for the daily cases of COVID-19 showed a peak on 26 January 2021. In Italy, different findings were reported in which the second wave began in August 2020 and continued to February 2021.^20^ A study in Spain demonstrated that the second wave started on 01 July 2020 and ended on 15 October 2020, indicating that this period was different to what was obtained in our study.^21^ In India, the peak of cases was observed around 01 March 2021.^19^ These differences could be a result of differences in geographical locations and climatic conditions across the globe.^22,23,24^

This study found a statistically significant difference in positivity rate between female travellers compared with male travellers, with female travellers more likely to test positive. These findings are consistent with those in Nigeria, where more asymptomatic female individuals than male tested positive.^17^ Other studies have also reported similar findings in which female individuals had higher odds of positivity than male individuals.^25,26^ This could be due to female patients having a higher health-seeking behaviour than male patients.^25^ In a study done in Netherlands, on data collected from March 2020 to August 2020, there was no significant difference in positivity rates between female patients and male patients.^27^ The study also found the age between 19 to 50 years to have a higher positivity rate compared to those who were 18 years or younger and those older than 50 years. This finding was not different from the study done in Wuhan, China, and Bahrain, Ireland, that reported a higher prevalence of COVID-19 in individuals who were less than 45 years old in Wuhan, and 20 to 49 years in Bahrain.^28,29^ This could be because those aged 18 years and younger were less susceptible to COVID-19 during the second wave.^30^ In addition, control measures such as closure of school, colleges and universities may have contributed to the age group 18 years and younger having a low positivity rate.^31^ On the other hand, most of the individuals older than 50 years were symptomatic and prone to hospitalisation as compared to younger individuals.^32^

### Limitations

This study had some limitations, one of which was that there was no control on the variables as this was a retrospective analysis of previously collected data. Findings in this study may not be generalisable, as the data were obtained from one testing site. However, the study had good power and the results are a true reflection of the country’s positivity rate. A prospective study with more variables is recommended.

### Conclusion

The positivity rate was found to be 16.0%, implying that there was continued community transmission despite the instituted public health guidelines. Age was not a predictor of a testing positive for COVID-19, whereas the month in which a test was done, the sex of the individual and their place of residence were good predictors. The positivity rate reported in this study suggests the need to heighten testing of SARS-CoV-2 among asymptomatic individuals.
